# Observers Efficiently Extract the Minimal and Maximal Element in Perceptual Magnitude Sets: Evidence for a Bipartite Format

**DOI:** 10.1177/09567976231223130

**Published:** 2024-01-18

**Authors:** Darko Odic, Tyler Knowlton, Alexis Wellwood, Paul Pietroski, Jeffrey Lidz, Justin Halberda

**Affiliations:** 1 Department of Psychology, University of British Columbia; 2 Department of Psychology, University of Pennsylvania; 3 School of Philosophy, University of Southern California; 4 Department of Philosophy, Rutgers University; 5 Department of Linguistics, University of Maryland, College Park; 6 Psychological and Brain Sciences, Johns Hopkins University

**Keywords:** representational format, computational theory of mind, perceptual magnitudes, working memory limits, open data, open materials, preregistered

## Abstract

The mind represents abstract magnitude information, including time, space, and number, but in what format is this information stored? We show support for the bipartite format of perceptual magnitudes, in which the measured value on a dimension is scaled to the dynamic range of the input, leading to a privileged status for values at the lowest and highest end of the range. In six experiments with college undergraduates, we show that observers are faster and more accurate to find the endpoints (i.e., the minimum and maximum) than any of the inner values, even as the number of items increases beyond visual short-term memory limits. Our results show that length, size, and number are represented in a dynamic format that allows for comparison-free sorting, with endpoints represented with an immediately accessible status, consistent with the bipartite model of perceptual magnitudes. We discuss the implications for theories of visual search and ensemble perception.

The computational theory of mind—the idea that the mind is an information-processing system akin to a Turing machine—is perhaps the most widely influential and generative idea about cognition (Anderson, 2009; Fodor, 1975; Gallistel & King, 2009; Pylyshyn, 1984; Turing, 2009). But if thinking is computing, what are the data structures that support this process? In other words, what is the *representational format* that permits some computations to be executed with ease while making others less transparent?

The study of representational formats has been especially tractable in perceptual magnitudes (i.e., representations of space, time, and number; e.g., Dehaene & Brannon, 2010; Feigenson et al., 2004; Gallistel, 1989; Odic & Starr, 2018; Pylyshyn, 2003). Imagine seeing a collection of fruit in a bowl. Vision represents many perceptual features, such as the location of each piece of fruit, their varied colors and sizes, total number, average size, and so on. But in what format is this information stored? Under one popular model, perceptual magnitudes are represented on a linear scale, with imprecision scaling proportionally with the value (Brannon et al., 2001; Gallistel, 2011; Gallistel & Gelman, 2000; Odic et al., 2016). For example, a series of neurons—each with a preferred value—collectively respond to the encoded value, with neurons further from the preferred line firing less, instantiating a tuning curve with scalar variability (Dehaene et al., 2003; Halberda & Odic, 2014; Harvey et al., 2013). Although such a format permits arithmetic operations to be transparently carried over to perceptual magnitudes, the need for dedicated feature detectors imposes computational demands because of the high dynamic range that the physically limited brain can represent. Given the high dynamic range of most perceptual magnitudes (e.g., at extremes, perception can represent brightness from even a few photons all the way to the brightness of staring directly at the sun), such a coding scheme must allocate an unrealistically high amount of neuronal real estate to represent any value along that range (Ala-Laurila & Rieke, 2014).

A common revision to this model is to distribute the feature detectors logarithmically (Dehaene, 2003; Dehaene et al., 2008). Small numbers of items would then be represented by dedicated feature detectors with high precision, whereas higher numbers would lack dedicated feature detectors and would be decoded from the collective and noisy combination of many neurons, leading to imprecision. But this revision does not permit transparent arithmetic operations because a log format could not represent the value of zero and would treat addition and subtraction as multiplication and division (Gallistel, 2011).

Gallistel (2011) proposed a solution that maintains transparent arithmetic with adequate compression—a bipartite format that codes magnitudes in two bits: the *value* and the *scale*. The first bit of information is the measurement value falling on an arbitrary scale (e.g., a value between 0 and 1). The second is a scaling variable that is determined by estimating the dynamic range—the difference between the smallest and the largest value in the current environment. Consider how computers represent very large numbers using the scientific notation: 5.1 × 10e^10^ is a bipartite format in which the exponent codes for the scale, whereas the other number specifies the interval (Gallistel, 2017). This allows the sensitivity of the internal representation to autoscale in accordance with the amount of information presented in the input, allowing for efficient storage over a wide dynamic range while maintaining transparent arithmetic operations over the stored values.

The bipartite model is theoretically appealing, but it has never been empirically tested in magnitude perception. We do so by investigating the identification of maximal and minimal elements in a set. Because the bipartite format’s autoscaling depends on the estimated dynamic range, it relies on the encoding of these endpoints with a high degree of precision; otherwise, it risks failing to appropriately scale the entire range of values. This implies that perceptual encoding mechanisms that extract magnitudes should—in early perceptual stages—code for the minimal and maximal values with a uniquely privileged status because every other value is coded in relation to them.

**Statement of Relevance**

How is information stored in the mind? Much like computers can store information as integers, strings, or floating points, the format of our mind’s representations dictates how information is used. With a population of college undergraduates, we show that a set of representations—perceptual magnitudes, including length, size, and number—are stored in a format that privileges endpoint values over inner ones. People can rapidly identify the maximal and minimal value in a set (e.g., find the longest or shortest line out of 11 heterogeneous options), even though they struggle to identify other members of the set (e.g., the third longest line). We propose that representations of perceptual magnitudes are in a bipartite format—akin to scientific notation— and therefore “scale” the representational set by the minimal and maximal value that we are currently attending to.

Returning to our bowl-of-fruit example, how might the visual system represent which of the fruits is “the largest” or “the smallest”? Under many models of perception and memory, this is a serial comparison problem: The observer must compare the size of each fruit to each other one through successive comparisons (Pashler & Badgio, 1985). This is analogous to how digital computers order values (e.g., the quicksort algorithm; for extensive review, see Cormen et al., 2022) but necessitates a mathematical limit to its efficiency: The time and memory demands grow with the size of the list because more comparisons must be done, predicting more errors as the set size grows. An alternative algorithm comes from ensemble perception and pooled-population normalization: Perception might preattentively code for the typical value in the entire set represented on a linear/log scale, with items that are further from the mean popping out as potential outliers via a similarity metric (Haberman & Whitney, 2012; Utochkin et al., 2023).

The bipartite format makes different predictions from both of these accounts. Unlike the sorting algorithm, it avoids the efficiency problem because the very act of representing a perceptual magnitude requires first encoding the minimal and maximal elements with high precision so that other values can be represented in relationship to the range. Therefore, the maximal and minimal elements are represented “for free” and do not require comparisons to each other value. Observers should therefore find these elements quickly and efficiently at any set size, so long as crowding or other low-level performance issues are kept to a minimum. Inner values, on the other hand, such as the second or third largest fruit, are not represented with this privileged status and would require successive comparisons, incurring a serial cost.

The bipartite format is also distinct from the similarity-to-the-mean account. Extracting the mean value (e.g., the average length line) improves with set size (Utochkin et al., 2023), predicting higher precision for finding the endpoints at higher set sizes. If the individual items are also equally spaced from each other, then increasing the set size also increases the range, again predicting that the maximal and minimal identification should improve with set size. The bipartite format, on the other hand, represents the range independently from the mean: It codes values from the range inward, not from the mean outward. It therefore—again—predicts no effect of set size.

In six experiments, we tested these hypotheses across a range of dimensions (length, area, and number). The first four experiments presented here are identical except for the stimuli used, and for simplicity we report them together as a group, noting differences between them where relevant. Experiments 1 and 2 examined performance on finding the longest and shortest lines in a set of five (Experiment 1) or seven (Experiment 2) lines. For Experiment 3, we asked participants to find the biggest and smallest set of dots, always presenting five sets but varying the number of individual dots in each set from one to five. Experiment 4 had participants find the most or least numerous set of dots. With the use of a within-subjects design, Experiment 5 returned to length perception and tested set sizes of seven, nine, and 11 lines. Finally, Experiment 6 controlled for a potential confound in the instructions provided to the participants.

## Open Practices Statement

All experiments in this article were preregistered (https://aspredicted.org/sn9xs.pdf), and all methods, stimuli, programs, and data are available on the OSF at https://osf.io/k6fqt.

## Experiments 1–4

### Participants

On the basis of pilot data, we determined that a minimum of 30 participants was sufficient to achieve power = 0.95 and α = 0.05. We preregistered participant exclusions with replacements if they did not complete the entire experiment or had at-chance performance; we also removed trials for which participants’ response times (RTs) were ± 3 *SD*s from their own mean. Participants were undergraduate students at the University of British Columbia, and course credit was given for participation. We did not collect demographic data on participants. Because we recruited participants online, we posted more sign-up slots than necessary to account for a higher rate of dropouts. In total we ended up collecting 36 participants for Experiment 1, 33 for Experiment 2, 57 for Experiment 3, and 39 for Experiment 4. For the reported analysis and to follow our preregistration plan, we report only on the first 30 nonexcluded participants in each experiment, but data for all other participants are available online, and all of our findings are replicated when the full sample is used for each experiment.

Participants signed up for a study via an institutional online sign-up sheet. During the first wave of recruitment, participants were randomly assigned to either Experiment 1 (five lengths) or Experiment 2 (seven lengths). During the second wave of recruitment, participants were all assigned to Experiment 3 (five single or average sizes), and during the final wave of recruitment all participants were assigned to Experiment 4 (five approximate numbers). These experiments were staggered for convenience given limitations of the institutional recruitment platform to test only one experimental protocol at a time. All research was approved by the University of British Columbia Office of Research Ethics.

### Method

The experiments were conducted online using the Pavlovia platform and PsychoPy Version 3 (Peirce et al., 2019). The code and stimuli for all experiments are available at https://osf.io/k6fqt. Participants completed a consent form and then followed a link to complete the task on their own computers.

#### Experiment 1 stimuli and procedure

The stimuli in Experiment 1 consisted of five lines drawn on the screen. Each line was drawn in a different color (yellow, blue, orange, green, and purple) at 80% opacity to allow them to be visible even if they intersected, effectively resembling pick-up sticks (lines were never permitted to nest within each other, however, because this would prevent object individuation). Because of variations in individual computers and screen resolutions during online testing, we report all values as a percentage of overall screen. To construct the sequence of lines, the longest line was first randomly chosen to be between 10% and 30% of the overall screen length (participants could therefore not memorize a specific length as any of the targets and could identify them only relative to the other values). Each successive line would then be shorter by a constant ratio—either 5%, 10%, 25%, or 50% shorter (e.g., if the first line was 100 pixels in the 10% condition, the second would be 90 pixels long, the third 81 pixels, the fourth 72 pixels, and so on). Because of this constant decrease in length, the middle line was the geometric average-length line in the set. Each line was placed in a random position within an invisible rectangular box bordering 80% of the screen on each side and was given a random orientation between 0° and 180°.

Before viewing the trial, participants were given a prompt asking them to identify the color of a specific line. In Experiment 1, participants were asked to identify the longest, second longest, third longest, second shortest, and shortest lines. Once the participant was ready to begin the trial, they clicked anywhere with the mouse. The lines then appeared for 2,000 ms and were then replaced by a series of colored rectangles, each corresponding to one of the colors presented during the previous trial. Participants clicked on the square they believed matched the color of the line they were asked to find.

The main dependent variables were accuracy (i.e., whether the participant selected the correct square) and RT (i.e., the time from stimulus offset to the click of the square). Each participant was asked about each ordinal position crossed with each ratio 10 times, yielding 200 total trials in Experiment 1 (five positions × four ratios × 10 trials). After completing the task, participants were debriefed and given course credit for their participation.

#### Experiment 2 stimuli and procedure

The stimuli and procedure in Experiment 2 were identical to those in Experiment 1 except that we increased the number of lines to seven, introducing a black and a white line. Participants were asked to identify the longest, second longest, third longest, fourth longest, third shortest, second shortest, and shortest lines. Each participant was asked about each ordinal position crossed with each ratio 10 times, yielding 280 total trials (seven positions × four ratios × 10 trials).

#### Experiment 3 stimuli and procedure

The stimuli were sets of circles that were equal in number but varied in color, individual area, and cumulative area. Each set was drawn from a set of five colors (yellow, blue, red, green, and purple). As above, we report the units in overall percentage of the screen to account for variability in screen size resulting from online testing. The cumulative area of the circles varied from 0.04% to 1.5% of the overall screen, again preventing participants from being able to memorize a specific value as any of the targets. Once the cumulative area of the circles was determined, each individual circle in that set was varied up to ± 20% of the average size, creating variability among the circles so that no single item was sufficient to determine the size of the set as a whole but preserving the overall cumulative area. To construct the sequence of sizes, the largest set was first randomly chosen to be between 0.2% and 1.5% of the overall screen in cumulative area. Each successive set would then be smaller by a constant ratio matching that of Experiment 1—either 5%, 10%, 25%, or 50% smaller. We also varied the total number of circles in each set to be either one, three, or five, with all sets having the same number. As noted above, this allowed us to examine whether performance is different for finding the largest circle (individual size) versus the largest circles (cumulative area). Each circle was placed in a random position within an invisible rectangular box bordering 80% of the screen on each side, and they were not segregated into sets by color. The circles were not allowed to overlap.

Before viewing the trial, participants were asked to identify the color of a specific circle or set of circles. For the one-dot trials, we asked participants to identify the biggest, second biggest, third biggest, second smallest, and smallest dots; for trials with three or five dots per set, we asked participants to identify the biggest, second biggest, third biggest, second smallest, and smallest dots. Once participants were ready to begin the trial, they clicked anywhere with the mouse. As in Experiment 1, the circles then appeared for a total of 2,000 ms, and participants then clicked on the rectangular box they believed matched the color they were asked to find. Once again, the key dependent variables were accuracy and RT, and participants completed 200 total trials.

#### Experiment 4 stimuli and procedure

The stimuli were colored dots spatially separated into five groups that varied in color (yellow, blue, red, green, and purple) and number. The smallest set of dots could have four dots, and the largest set of dots could have 40 dots (this was effectively the largest range we could make to fit the total number of dots on the screen). Each dot took approximately 0.05% of the overall screen size but varied randomly ± 20% to introduce some variability. To construct the sequence of numbers, the most numerous set was first randomly chosen to be between 20 and 40 dots to prevent memorization of target values, and each successive set would then be reduced in number by a constant ratio matching that of Experiment 1—either 5%, 10%, 25%, or 50% fewer. We clustered the dots of the same set together because we found that observers could not otherwise clearly identify the sets by color alone if they were fully intermixed. Because average item size was equivalent across the sets, item size was not a cue to the correct answer; similarly, because each set was drawn within an equally sized area, convex hull was not a cue to the answer (although density positively covaried with number). For our current research questions, participants who answered on the basis of density (or any other feature of these collections) would be just as interesting as responses based on number because these alternative dimensions would also be codable on a bipartite format.

Before viewing the trial, participants were asked to identify the set with the most, second most, third most, second fewest, and fewest dots. Once participants were ready to begin the trial, they clicked anywhere with the mouse. As in Experiment 1, the stimuli then appeared for a total of 2,000 ms, and participants then clicked on the square they believed matched the color they were asked to find. Once again, the key dependent variable was accuracy and RT, and participants completed 200 total trials.

### Analysis plan

Our primary analysis of interest was a comparison of accuracy and RT across probed positions. The main prediction of the bipartite account is that the outer positions—the minimum and the maximum—should have privileged status, so questions probing them should be high in accuracy and low in RT, with performance getting worse with each successive position away from the edges. The bipartite account also predicts that there should be no effect of set size. On the other hand, the “sorting” account predicts that participants should—especially given higher memory constraints—perform at chance or equivalently poorly for all positions because identifying any position requires a minimum of four to six successive comparisons, and the similarity-to-the-mean account predicts that performance should improve with higher set sizes. We made no predictions about differences across the three dimensions tested (length, area, and number) and expected the patterns to hold across all of them given that perceptual magnitudes are theorized to be coded in a similar format (Cantlon et al., 2009; Dehaene & Brannon, 2010; Walsh, 2003). We also made no predictions about the effect of ratio because smaller ratios would naturally make all nearby values more confusable (e.g., at the smallest ratio, the longest and the second longest line would be similar enough in length that quickly identifying the color may be more difficult under time pressure).

### Results

#### Experiment 1

**Fig. 1.**
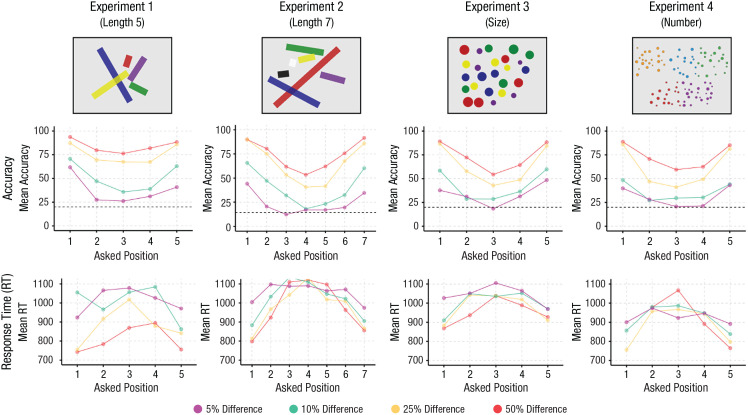
Results from Experiments 1 through 4. The top row shows example images from the four experiments. Note that the presented lines were actually thinner and presented more foveally than illustrated here. The middle row shows the accuracy across Position × Ratio. The dashed line indicates at-chance performance. The bottom row shows response times across Position × Ratio.

A 5 (Position) × 4 (Ratio) repeated-measures analysis of variance (ANOVA) with accuracy as the dependent variable showed a significant main effect of position, *F*(4, 116) = 68.85, *p* < .001, *f =* 1.54, a significant main effect of ratio, *F*(3, 87) = 208.67, *p* < .001, *f =* 2.68, and a significant interaction, *F*(12, 348) = 4.07, *p <* .001, *f* = 0.37. As can be seen in Figure 1, participants performed above chance at all positions but were especially excellent at finding the longest and shortest line in the set at any ratio, with a serial drop-off in performance with each line as they approached the middle (i.e., the third longest line). Tukey’s honestly significant difference (HSD) tests revealed significant differences between all pairwise positions (all *ps* < .001) except Position 2 versus Position 3, *t*(116) = 2.28, *p* = .16, Position 2 versus Position 4, *t*(116) = .574, *p* = .98, and Position 3 versus Position 4, *t*(116) = −1.712, *p* = .43. Although the bipartite format does not predict the significant difference between Positions 1 and 5 (i.e., longest vs. shortest), there is a long-standing finding that negative poles are slower and less accurate than positive ones (Klatzky et al., 1973; Odic et al., 2013). We return to this issue in the General Discussion section because we observed this significant difference only between the minimum and the maximum in Experiment 1.

Performance decreased linearly with ratio, with the worst performance at 5% difference, *M* = 37.4%, 95% confidence interval (CI) = [32.4, 42.4], and the best performance at 50% difference, *M* = 84.0%, 95% CI = [79.0, 89.0]. The significant interaction resulted primarily from the general flattening of performance as the ratio became harder. For example, at a difference of 5%, we found significant differences only between Position 1 versus Position 2, *t*(116) = 3.81, *p* = .002, Position 1 versus Position 3, *t*(116) = 4.77, *p* < .001, and Position 1 versus Position 4, *t*(116) = 3.22, *p =* .012.

This pattern of performance was not driven by a speed-accuracy trade-off: A 5 (Position) × 4 (Ratio) repeated-measures ANOVA with RT as the dependent variable showed a significant main effect of position, *F*(4, 116) = 26.22, *p* < .001, *f* = 0.95, a significant main effect of ratio, *F*(3, 87) = 54.17, *p* < .001, *f* = 1.37, and a significant interaction, *F*(12, 348) = 2.091, *p* = .017, *f* = 0.27, with participants faster to find the shortest and longest lines, especially at the easiest ratios. In other words, participants were most accurate and fastest for finding the longest and shortest lines and showed a progressive decrease in accuracy and increase in RT with each successive position.

#### Experiment 2

A 7 (Position) × 4 (Ratio) repeated-measures ANOVA with accuracy as the dependent variable showed a significant main effect of position, *F*(6, 174) = 111.0, *p* < .001, *f* = 1.96, a significant main effect of ratio, *F*(3, 87) = 214.6, *p* < .001, *f =* 2.72, and a significant interaction, *F*(18, 522) = 5.55, *p <* .001, *f =* 0.44. Performance was again overall best for the longest and shortest positions, with a successive decrease in performance toward the middle position. Tukey’s HSD tests revealed significant differences between all pairwise positions (all *ps* < .02) except Position 1 versus Position 7, *t*(174) = 2.05, *p* = .38, Position 3 versus Position 5, *t*(174) = 1.621, *p* = .662, and Position 4 versus Position 5, *t*(174) = −1.897, *p* = .48. Performance decreased linearly with ratio, with the worst performance at 5% difference, *M* = 23.6%, 95% CI = [18.9, 28.3], and the best performance at 50% difference, *M* = 73.1%, 95% CI = [68.4, 77.8]. Even at this level of difficulty, however, performance was above chance. As in Experiment 1, the significant interaction resulted primarily from participants having difficulty identifying the ordinal position of any line as the ratio became harder (Fig. 1).

We again did not find the advantage for the outer positions to be a speed-accuracy trade-off. A 7 (Position) × 4 (Ratio) repeated-measures ANOVA with RT as the dependent variable showed a significant main effect of position, *F*(6, 174) = 2.985, *p* = .008, *f* = 0.32, a significant main effect of ratio, *F*(3, 87) = 6.02, *p* < .001, *f* = 0.46, and a nonsignificant interaction, *F*(18, 522) = 0.747, *p* = .763, *f* = 0.16, with participants again faster to find the shortest and longest lines.

Finally, we examined whether the increase in the number of lines between Experiments 1 and 2 produced any change in accuracy for the longest and shortest lines (we did not compare the inner positions because these differed across the two experiments). A 2 (Experiment) × 2 (Position) mixed-measure ANOVA showed no main effect of experiment, *F*(1, 58) = 1.597, *p* = .21, *f =* 0.17, a significant main effect of position, *F*(1, 58) = 43.54, *p* < .001, *f* = 0.87, and a significant interaction, *F*(1, 58) = 4.71, *p* = .034, *f =* 0.28. The interaction, however, was primarily driven by the lack of difference between the longest and shortest lines in Experiment 2 and the presence of that difference in Experiment 1. Indeed, there was no significant difference in accuracy between finding the longest line across the two experiments, *t*(72) = 1.91, *p* = .23, or for finding the shortest line, *t*(72) = 0.479, *p =* .96. Therefore, the increase in the number of lines from five to seven did not affect performance for the two outer positions—exactly as expected from the bipartite format but contrary to both an item-based sorting mechanism (which would have required additional comparisons given seven as opposed to five lines) and the comparison to the mean mechanism (which would have predicted better performance with seven lines because the longest and shortest lines are further away from the mean).

#### Experiment 3

A 7 (Position) × 4 (Ratio) × 3 (Dots in Set) repeated-measures ANOVA with accuracy as the dependent variable showed a significant main effect of position, *F*(4, 116) = 102.60, *p* < .001, *f* = 1.88, a significant main effect of ratio, *F*(3, 87) = 194.3, *p* < .001, *f =* 2.59, a significant main effect of dots in set, *F*(2, 58) = 15.32, *p* < .001, *f* = 0.73, a significant Position × Ratio interaction, *F*(12, 348) = 6.28, *p* < .001, *f* = 0.47, a significant Position × Dots in Set interaction, *F*(8, 232) = 6.54, *p* < .001, *f* = 0.47, a significant Ratio × Dots in Set interaction, *F*(6, 174) = 4.824, *p* < .001, *f =* 0.41, and a significant three-way interaction, *F*(24, 696) = 3.575, *p* < .001, *f* = 0.35.

As with length, we found that performance was again overall best for the biggest and smallest positions, with a successive decrease in performance toward the middle position. Tukey’s HSD tests revealed significant differences between all pairwise positions (all *ps* < .001) except Position 1 versus Position 5, *t*(116) = −1.18, *p* = .80, and Position 2 versus Position 4, *t*(116) = 1.258, *p* = .717. Performance decreased linearly with ratio, with the worst performance at 5% difference, *M* = 33.2%, 95% CI = [29.8, 36.5], and best performance at 50% difference, *M* = 73.5%, 95% CI = [70.2, 76.9]. Once again, even the worst-performing condition was above chance. We found better performance overall when each set consisted of a single dot, *M* = 57.8%, 95% CI = [54.8, 60.8], than with three dots, *M* = 50.6%, 95% CI = [47.5, 53.6], or five dots, *M* = 50.4%, 95% CI = [47.4, 53.5], and, as in earlier experiments, performance flattened as the ratio became more difficult. Most importantly, we found an advantage for the biggest and smallest position at every ratio and with any number of dots, showing that the observed advantage for the endpoints generalizes from individuals to ensembles.

We found the same pattern for RTs: A 7 (Position) × 4 (Ratio) × 3 (Dots in Set) repeated-measures ANOVA with RT as the dependent variable showed a significant main effect of position, *F*(4, 116) = 21.17, *p* = < .001, *f* = 0.87, a significant main effect of ratio, *F*(3, 87) = 15.73, *p* < .001, *f* = 0.74, a significant main effect of dots in set, *F*(2, 58) = 5.29, *p* = .008, *f =* 0.43, a significant Position × Ratio interaction, *F*(12, 348) = 2.046, *p* = .012, *f =* 0.27, a nonsignificant Position × Dots in Set interaction, *F*(8, 232) = 1.232, *p* = .281, *f =* 0.21, a nonsignificant Ratio × Dots in Set interaction, *F*(6, 174) = 0.456, *p* = .84, *f =* 0.13, and a nonsignificant three-way interaction, *F*(24, 696) = 1.357, *p* = .12, *f =* 0.22. Performance was fastest for the biggest and smallest positions compared with the inner positions, with no significant difference between the outer two positions, *t*(116) = −1.417, *p* = .62, and was slightly faster for sets with one dot, *M* = 970, *SE* = 30.60, 95% CI = [907, 1,032], compared with three dots, *M* = 1,015, *SE* = 30.6, 95% CI = [953, 1,077], and five dots, *M* = 1,004, *SE* = 30.6, 95% CI = 942, 1,066].

#### Experiment 4

A 7 (Position) × 4 (Ratio) repeated-measures ANOVA with accuracy as the dependent variable showed a significant main effect of position, *F*(4, 116) = 91.49, *p* < .001, *f* = 1.78, a significant main effect of ratio, *F*(3, 87) = 249.0, *p* < .001, *f =* 2.93, and a significant interaction, *F*(12, 348) = 6.159, *p <* .001, *f =* 0.46. Performance overall was again best for the most and the fewest positions, with a successive decrease in performance toward the middle position. Tukey’s HSD tests revealed significant differences between all pairwise positions (all *ps* < .04) except Position 1 versus Position 5, *t*(116) = 1.167, *p* = .77, Position 2 versus Position 4, *t*(116) = 1.357, *p* = .65, and Position 3 versus Position 4, *t*(116) = −1.479, *p* = .578. Performance decreased linearly with ratio, with the worst performance at 5% difference, *M* = 30.60%, 95% CI = [26.6, 36.6], and best performance at 50% difference, *M* = 73.4%, 95% CI = [69.3, 77.4]. Once again, performance on even the worst-performing question was above chance. As in Experiment 1, the significant interaction resulted primarily from participants having difficulty identifying the ordinal position of any line as the ratio became harder (Fig. 1).

We again did not find the advantage for the outer positions to be a speed-accuracy trade-off. A 7 (Position) × 4 (Ratio) repeated-measures ANOVA with RT as the dependent variable showed a significant main effect of position, *F*(4, 116) = 16.45, *p* < .001, *f* = 0.75, a nonsignificant main effect of ratio, *F*(3, 87) = 1.752, *p* = .16, *f* = 0.25, and a nonsignificant interaction, *F*(12, 348) = 1.421, *p* = .154, *f* = 0.22, with participants again faster to find the shortest and longest lines.

### Discussion

In four experiments ranging across three dimensions (length, size, and number), across individual and ensemble perception, and across sets of five and seven—we found a consistent advantage for identifying the maximal and minimal element in an array and a steady decrease in performance, with each successive ordinal position away from the maximum and minimum. Therefore, we have evidence that the endpoints of perceptual magnitudes are preferentially and efficiently coded compared with other values.

These results broadly replicate patterns in visual searches showing an advantage for finding predefined targets (e.g., a 1- × 1-in. square) when those objects are also coincidentally the largest or smallest items in the set (e.g., Becker et al., 2013; Treisman & Gelade, 1980; Wolfe, 2021). Unlike these past paradigms, (a) we did not have target values that could be preselected through attentional tuning because the value of the objects changed on every trial, (b) our paradigm was maximally heterogeneous in that every object was distinct from every other one, and (c) we found no cost in accuracy or RT for increasing the set size. Because the bipartite format is a theory of how features are represented, it can be considered an extension on work in visual search, providing a mechanism for why attention can be easily directed toward the endpoints of a scale—these values are inherently meaningful in the bipartite format because magnitude perception proceeds from identifying the range and then coding each other object within that scale.

Experiments 1 and 2 provide evidence against a set-size effect, but they do so in a between-subjects design and only for set sizes of five and seven. To demonstrate that observers are not finding the endpoints from comparing each item to the mean value, we would ideally show that set-size effects do not occur even as we extend the range even further. In Experiment 5, therefore, we asked participants to find either the longest-, shortest-, or middle-length line in set sizes of seven, nine, and 11. Because the ratio between each successive line was held constant in our stimuli, the difference between the longest/shortest line and the mean-length line increased, on average, by a ratio of 1.7 at a set size of seven, 2.0 at a set size of nine, and 2.5 at a set size of 11. This stimulus structure also changed the standardized difference: The *z* score of the longest line increased with set size (from approximately 1.75 at a set size of seven to approximately 2.20 at a set size of 11), whereas the shortest line decreased with set size (from approximately −1.00 to approximately −0.85). Models predicting that the longest/shortest line is found through a comparison to the mean—either via a ratio or a standardized-difference metric (Robitaille & Harris, 2011; Utochkin et al., 2023)—would therefore predict a set-size effect. The bipartite format, on the other hand, predicts no effect of set size.

## Experiment 5

### Participants

We collected data from 32 participants, two of whom were excluded for not completing the task, leaving us with 30 participants in the final sample. Participants were recruited in the same manner as Experiments 1 through 4 and tested online via PsychoPy Version 3. The method and data from all 30 participants are available online at https://osf.io/k6fqt.

### Stimuli and procedure

To accommodate set sizes of up to 11 lines, we had to make two changes to the procedure. First, all of the lines were colored white on a gray background. After displaying the lines for 2,000 ms, the lines were replaced with small, identically sized dots at the center of each of the lines. Participants were asked to use the mouse and click on the dot that matched the position of the line they were asked to find (i.e., either the longest/middle/shortest line). This method also allowed us to directly compare RTs across set sizes, which would have been difficult with the color task given that participants would have to spend more time searching for the correctly colored box with higher set sizes. Second, to accommodate the higher number of trials participants needed to complete, we set the ratio of every trial to 1.20. As discussed above, this stimulus setup implies that the endpoints increasingly diverge from the mean as the set size increases.

At the start of each trial, participants were asked to search for either the longest, middle, or shortest line. After pushing a button to start, they would see a display of either seven, nine, or 11 lines on the screen, followed by a replacement by dots that they could click on. Participants completed 3 (Position) × 3 (Set Size) × 23 repetitions for a total of 207 trials.

### Results

**Fig. 2.**
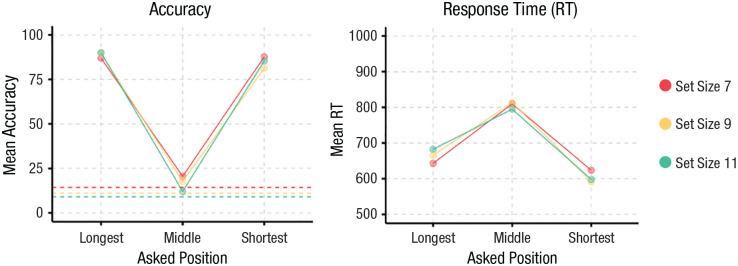
Results from Experiment 5. Accuracy over Position × Set Size is shown on the left (chance performance differs by set size and is indicated by individual dashed lines). Response times over Position × Set Size are shown on the right.

A Position × Set Size repeated-measures ANOVA with accuracy as the dependent variable showed a significant main effect of position, *F*(2, 58) = 517.05, *p* < .001, *f* = 4.22, a marginal effect of set size, *F*(2, 58) = 3.09, *p* = .053, *f =* 0.33, and a significant Position × Set Size interaction, *F*(4, 116) = 6.70, *p* < .001, *f =* 0.48. As seen in Figure 2, post hoc contrasts reveal that the main effect of question was driven by a significant difference between the longest and middle positions, *t*(58) = 28.64, *p* <.001, and shortest and middle positions, *t*(58) = −26.98, *p* < .001, and no difference between the longest and shortest positions, *t*(58) = 1.67, *p* = .23. The significant interaction was driven by a linear trend for performance getting worse with set size for the middle position, β = −2.14, 95% CI = [−3.24, −1.04], in contrast to flat slopes with set size for the longest, β = 0.76, 95% CI = [−0.34, 1.86], and shortest, β = −0.58, 95% CI = [−1.68, 0.52], positions. The negative slope for the middle position replicates work in visual search showing that endpoint targets are easier to find than middle targets (Treisman & Gelade, 1980; Wolfe, 2021). The lack of any such effects for the endpoints shows a clear violation of the comparison from the mean models, which predict better performance with increasing set size.

We also examined RTs. A Position × Set Size repeated-measures ANOVA with RT as the dependent variable showed a significant main effect of position, *F*(2, 58) = 104.0, *p* < .001, *f* = 1.89, and no effect of set size, *F*(2, 58) = 0.02, *p* = .98, *f =* 0.02, or a significant Position × Set Size interaction, *F*(4, 116) = 1.81, *p* = .13, *f =* 0.25. The significant main effect was driven by the middle position being significantly slower than the longest position, *t*(58) = −9.97, *p* < .001, which was in turn slower than the shortest position, *t*(58) = −4.04, *p* < .001. We found no significant set-size effects or interactions, suggesting that neither a serial nor capacity-limited parallel search model could account for our data.

### Discussion

Recall that participants in Experiments 1 through 4 were instructed to find a position that was *X* places from the maximal or minimal element (e.g., third longest line). Thus, an alternative explanation might suggest that our instructions signaled to them to first identify the maximal or minimal element and then proceed from there to the asked position, forcing them to represent the endpoints with additional attention or precision to begin the search process for the other values. This possibility can be easily tested by changing the instructions and instead asking participants to find the *X* position from the middle (e.g., “Which line is second above the middle line?”). If the position named in the instructions provides an anchor value from which comparisons begin, then we should now find that participants are best with the middle position and progressively worse as they approach the outer edges.

## Experiment 6

### Participants

As with Experiments 1 through 4, we aimed to recruit 30 participants. We ended with a sample of 32 participants, two of whom were excluded for not completing the task, leaving us with 30 participants in the final sample. Unlike the first four experiments, all participants were recruited via Amazon Mechanical Turk. Recruitment criteria included being U.S. citizens, having completed at least 100 human-intelligence tasks (HITs), and having at least a 95% approval rating for completed HITs. The data from all 30 participants is available online at https://osf.io/k6fqt.

### Stimuli and procedure

The stimuli were identical to those in Experiment 2—sets of seven colored lines presented on the screen for 2,000 ms followed by a series of colored squares that participants would click to indicate the correct answer. The only difference was that the software used was hosted on Heroku instead of Pavlovia (note that this changed the data format uploaded online for this experiment compared with the others) and that participants were asked a different set of questions: “Which line is the third/second/first above/below the middle?” or “Which line is the middle?” The key dependent variables were accuracy and RT, and participants completed 200 total trials.

### Results

**Fig. 3.**
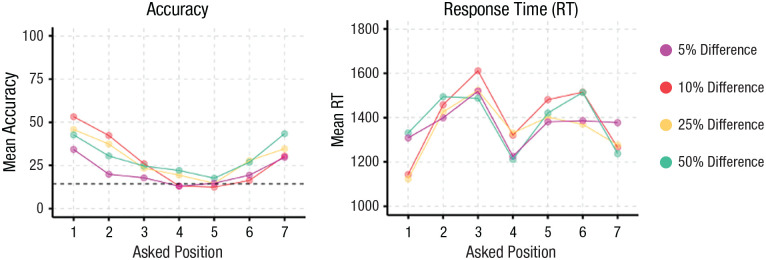
Results from Experiment 6. Accuracy over Position × Ratio is shown on the left, with the dashed line indicating at-chance performance. Response times over Position × Ratio are shown on the right.

A 7 (Position) × 4 (Ratio) repeated-measures ANOVA with accuracy as the dependent variable showed a significant main effect of position, *F*(6, 174) = 16.78, *p* < .001, *f* = 0.76, a significant main effect of ratio, *F*(3, 87) = 7.04, *p* < .001, *f =* 0.49, and a significant interaction, *F*(18, 522) = 3.127, *p <* .001, *f =* 0.33. As can be seen in Figure 3, overall performance was clearly worse, on average, than in Experiment 2 (we suspect because of testing on Mechanical Turk with a more heterogeneous sample), but there was still a clear benefit for the maximal and minimal positions, with a successive decrease in performance toward the middle position, even though the middle question was the linguistic anchor. The most relevant finding for our hypothesis was that there were significant differences for all pairwise comparisons involving the maximal and minimal positions (all *p*s < .03) except for Position 1 versus Position 7, *t*(174) = 2.622, *p* = .13, and Position 2 versus Position 7, *t*(174) = −0.558, *p* = .99; none of the inner positions were significantly different from each other. Performance was lower at 5% difference, *M* = 21.2%, 95% CI = [15.0, 27.5], but similar at the other three ratios. The significant interaction again resulted from participants having difficulty identifying the ordinal position of any line as the ratio became harder (Fig. 3).

A 7 (Position) × 4 (Ratio) repeated-measures ANOVA with RT as the dependent variable showed a significant main effect of position, *F*(6, 174) = 16.78, *p* < .001, *f* = 0.76, a significant main effect of ratio, *F*(3, 87) = 7.04, *p* < .001, *f* = 0.49, and a significant interaction, *F*(18, 522) = 3.127, *p* < .001, *f* = 0.33. There was still an RT advantage for the outer-edge items; however, unlike Experiment 2, we also found faster performance for the middle position (Fig. 3). Therefore, although the linguistic anchor did not have an effect on accuracy, it did have an effect on RTs; critically, however, the advantage for the outer-edge items was still preserved for both accuracy and RT.

## General Discussion

In six experiments, we demonstrated that (a) participants can easily and quickly identify the endpoint elements in a set for length, size, and number representations, especially when compared with the inner ordinal positions (Experiments 1–6); (b) participants’ ability to find the maximal and minimal element was not affected by increasing the number of sets (Experiments 1, 2, and 5) or by increasing the number of items within a set (Experiments 3 and 4); and (c) these findings did not result from alternative explanations such as a speed-accuracy trade-off (Experiments 1–4) or the linguistic anchor provided in the instructions (Experiment 6). Our results show that participants can identify the endpoints in a set of perceptual magnitude values without sorting because such an algorithm would predict a decrease in performance as the number of comparisons grew (Pashler & Badgio, 1985). They also show that participants can identify the endpoints without relying on a similarity metric that compares each item to the mean value because the distance between the endpoints and the mean (either in ratio or standardized-difference space) did not improve performance (Experiment 5). Instead, perceptual magnitude endpoint values are privileged and easily extracted from the visual scene even when not directly named (Experiment 6). The inner values, however, show a cost that is consistent with successive comparison, using something akin to the quicksort algorithm.

Our results are consistent with a representational format for perceptual magnitudes that privileges the endpoints over the inner values. Neither the log nor the linear-coding models predict this pattern because they posit no reason to privilege the endpoints over other values: Any value represented on these scales is equal to any other, and—especially when all the values are highly heterogeneous—any ordinal position must be identified through an algorithm akin to successive comparison. Our results are most consistent with the bipartite model of perceptual magnitudes proposed by Gallistel (2011), who coded for magnitudes on an arbitrary scale that was scaled according to the dynamic range of the input. Under this model, each value is represented relative to the endpoints (i.e., autoscaled to the minimum/maximum), and the coding of values begins from the range inward.

Our results are consistent with and extend work by Pashler and Badgio (1985), who found that participants can identify the highest Arabic digit in sets of two to six digits. This finding was explained by a process that places Arabic digits on a mental number line followed by a search for the value that is “rightmost” on the line. Assuming—as has been shown in previous work—that Arabic digits are themselves automatically mapped to a perceptual number scale (Dehaene et al., 2003; Fias et al., 2003; Odic & Starr, 2018), our findings support and extend this prior work. Rather than assuming that the mental number line is infinite and that participants search for the rightmost position, autoscaling in the bipartite format predicts that participants should find the highest (or, although untested, the lowest) Arabic digit so long as the format is mapped to a bipartite perceptual magnitude. A prediction for future work would be that participants who either do not possess this mapping (e.g., young children) or have lost it (e.g., because of brain damage) should show the endpoint benefit for displays of dots but not for Arabic digits.

Our work also extends findings in visual search and short-term memory that show that observers are sensitive to relative changes in targets versus distractors (Becker, 2010; Becker et al., 2013; Martin & Becker, 2021). For example, in change-detection tasks, color changes from endpoints to inner values (e.g., changing the reddest square) are sometimes more noticeable than changes from inner-to-inner positions. Likewise, searches for predefined targets that are near the middle of the scale appear to proceed from the endpoints toward inner values, even when the endpoints are never targets themselves (Hamblin-Frohman et al., 2023). We propose that the bipartite format explains how features are coded before attention operates over them in memory and visual-search tasks. A change in the endpoint requires an update in autoscaling that may be a reliable attentional cue that explains change-detection effects. And endpoints are easily accessible anchors from which features are represented in the bipartite model, such that participants might easily define attentional templates for other targets relative to them, as shown by the work of Hamblin-Frohman and colleagues.

As discussed above, a benefit for predefined targets that are incidentally also endpoint values has been previously demonstrated in the visual-search literature (for review, see Wolfe, 2021). The bipartite format provides a mechanistic explanation for both when we should and should not expect visual search to benefit from endpoint targets. Because feature endpoints are privileged in the bipartite format, any visual-search task that uses targets defined on a bipartite dimension should produce benefits for the maximal and minimal values as targets. But because not all perceptual dimensions are expected to be in the bipartite format (e.g., orientation is a circular dimension that does not require dealing with infinity), we should not observe endpoint effects in all dimensions. This is consistent with the work of Wolfe et al. (1992), who showed that the steepest line is not found faster in heterogeneously oriented sets. Our results, therefore, intersect with the rich literature on visual search by providing a plausible format for the dimensional-feature scales over which attention can be tuned and targets can be selected.

Potential limits on the generalizability of our findings include the population sampled (college undergraduates) and that our stimuli were artificial compared with real-world magnitude perception and presented in a highly controlled environment.

## Transparency

*Action Editor:* Krishnankutty Sathian

*Editor:* Patricia J. Bauer

*Author Contributions*

**Darko Odic:** Conceptualization; Data curation; Formal analysis; Funding acquisition; Investigation; Methodology; Project administration; Resources; Software; Supervision; Validation; Visualization; Writing – original draft; Writing – review & editing.

**Tyler Knowlton:** Conceptualization; Data curation; Formal analysis; Investigation; Methodology; Software; Validation; Writing – original draft; Writing – review & editing.

**Alexis Wellwood:** Conceptualization; Investigation; Methodology; Validation; Visualization; Writing – review & editing.

**Paul Pietroski:** Conceptualization; Investigation; Methodology; Validation; Writing – original draft; Writing – review & editing.

**Jeffrey Lidz:** Conceptualization; Funding acquisition; Investigation; Methodology; Project administration; Resources; Supervision; Validation; Writing – review & editing.

**Justin Halberda:** Conceptualization; Funding acquisition; Investigation; Methodology; Project administration; Resources; Supervision; Validation; Writing – review & editing.

**Open Practices:** This article has received the badges for Open Data, Open Materials, and Preregistration. More information about the Open Practices badges can be found at http://www.psychologicalscience.org/publications/badges.
